# Oral-Systemic Links: A Narrative Review of the Role of Periodontitis in Alzheimer’s Disease Development

**DOI:** 10.7759/cureus.109402

**Published:** 2026-05-21

**Authors:** Nikolaos Spantidakis, Vasileios Zisis, Christina Charisi, Filippos Fytros, Konstantinos Poulopoulos, Thomas Chontos, Andreas Yiannouras, Petros Papadopoulos, Asterios Katsagkolis, Vasiliki Arsoudi, Zoi Maria Thomaidi, Athanasios Poulopoulos, Smaragda Diamanti

**Affiliations:** 1 Dentistry (Oral Medicine-Oral Pathology), European University Cyprus, Nicosia, CYP; 2 Oral Medicine/Pathology, Aristotle University of Thessaloniki, Thessaloniki, GRC; 3 Pediatric Dentistry, Aristotle University of Thessaloniki, Thessaloniki, GRC; 4 Dentoalveolar Surgery, Surgical Implantology and Radiology, Aristotle University of Thessaloniki, Thessaloniki, GRC; 5 First University Clinic of Ophthalmology, General Hospital of Athens "G. Gennimatas", Athens, GRC

**Keywords:** alzheimer’s dementia, alzheimer’s-related disease, chronic generalized periodontitis, oral-systemic connection, periodontal status

## Abstract

Alzheimer’s disease (AD) and periodontitis are prevalent chronic conditions that disproportionately affect aging populations and pose substantial public health challenges worldwide. Increasing evidence suggests a potential association between these two diseases, with chronic oral infection and systemic inflammation emerging as key linking mechanisms. Periodontitis is characterized by a dysbiotic oral microbiome and persistent inflammatory responses that can lead to the dissemination of periodontal pathogens and their virulence factors into the systemic circulation. Notably, some studies have reported the detection of pathogens such as *Porphyromonas gingivalis* and their toxic products in the brains of individuals with AD, implicating a possible role in neuroinflammation and neurodegeneration. However, it should be clarified that detection does not establish causation. This narrative review aims to synthesize the existing evidence from animal studies exploring the link between periodontitis and AD and its related mechanisms, including neuroinflammation, amyloid and tau pathology, blood-brain barrier dysfunction, and systemic interactions. The electronic search in PubMed yielded 585 results. We focused on the past 10 years, thus removing 114 results. A total of 471 studies remained. Of the 471 articles reviewed, 239 studies were excluded based on their titles, abstracts, publication types, and topics because of inappropriate study designs (i.e., designs other than cross-sectional or animal studies). A total of 232 studies were further investigated. In this review, the analysis focused exclusively on animal studies, and the full texts were assessed against predefined eligibility criteria focusing on study design, animal model, periodontal exposure, and AD-related outcomes. Studies that met all inclusion criteria were included, whereas articles with inappropriate study designs or irrelevant outcomes were excluded. After full-text screening, 101 studies remained. Preclinical (animal) evidence supported plausible mechanistic links between periodontitis and AD. Furthermore, oral pathogens appear to mediate this ongoing neuroinflammation.

## Introduction and background

Alzheimer’s disease (AD) is a neurodegenerative disorder that progressively worsens and is associated with cognitive impairment, memory loss, and the development of characteristic pathological changes such as amyloid-β plaques and neurofibrillary tangles. Despite extensive research, the etiology of AD remains multifactorial and incompletely understood. The role of chronic inflammation [1,2] and peripheral infections in the development of AD has increasingly become a focus of interest [3,4].

Periodontitis is a chronic inflammatory disorder of the periodontal tissues caused mainly by dysbiotic oral microbiota [5]. *Porphyromonas gingivalis *has emerged as a significant pathogen among the implicated microorganisms because of its virulence factors, such as gingipains, lipopolysaccharides (LPS), and outer membrane vesicles (OMVs) [6-8]. In particular, it has been theorized that *P. gingivalis *can migrate from the oral cavity to the brain via the bloodstream, cross the blood-brain barrier (BBB), and thereby trigger neuroinflammation. Gingipains may act as neurotoxins and have been associated with the accumulation of amyloid-β plaques and tau protein damage.

Periodontitis has been associated with systemic conditions such as cardiovascular disease, diabetes, and, more recently, neurodegenerative disorders; however, the underlying mechanisms remain under investigation. Recent experimental studies indicate that periodontal pathogens and their virulence factors may affect CNS function through several mechanisms, including systemic inflammation, disruption of the BBB, possible bacterial translocation/invasion, and alteration of immune responses [3-5,9,10]. Moreover, emerging evidence suggests that the gut-brain axis and metabolic comorbidities may mediate these effects, indicating a multisystem interaction. Nevertheless, conflicting evidence, limitations of animal models, and challenges in translating preclinical findings to humans should be acknowledged.

Animal research offers a controlled approach for studying these mechanisms and allows detailed exploration of causality and pathophysiological pathways without the challenges associated with human studies. A review of the available literature may help synthesize and classify the key findings of animal studies, thereby organizing the evidence for readers. An increasing body of preclinical evidence suggests that periodontal disease may contribute to the development and progression of AD-like changes, although translational limitations should be considered [11-13]. This narrative review aims to synthesize the existing evidence from animal studies exploring the link between periodontitis and AD. In particular, the analysis focused exclusively on animal studies, including study design, animal model, periodontal exposure, and AD-related outcomes.

## Review

An extensive literature review was performed using the electronic database PubMed/MEDLINE to identify pertinent research on the interaction between periodontitis and AD. The search strategy involved the use of Medical Subject Headings (MeSH) and search keywords associated with periodontal disease, *Porphyromonas gingivalis*, Alzheimer disease, dementia, cognitive impairment, and amyloid-β. The following MeSH terms were applied: ((("Periodontal Diseases"[Mesh] OR "Periodontitis"[Mesh] OR periodontitis[Title/Abstract] OR "periodontal disease"[Title/Abstract] OR "Porphyromonas gingivalis"[Mesh] OR "Porphyromonas gingivalis"[Title/Abstract])) AND (("Alzheimer Disease"[Mesh] OR "Dementia"[Mesh] OR Alzheimer*[Title/Abstract] OR dementia[Title/Abstract] OR "cognitive impairment"[Title/Abstract] OR "amyloid beta"[Title/Abstract]))) AND (("Animals"[Mesh] OR "Animal Experimentation"[Mesh] OR mouse[Title/Abstract] OR mice[Title/Abstract] OR rat[Title/Abstract] OR rats[Title/Abstract] OR rodent*[Title/Abstract]) OR ("Cross-Sectional Studies"[Mesh] OR "cross-sectional"[Title/Abstract] OR cross sectional[Title/Abstract] OR prevalence[Title/Abstract])) NOT ("Systematic Review"[Publication Type] OR "Meta-Analysis"[Publication Type] OR review[Publication Type]).

The final electronic search was conducted on February 15, 2026. In total, the search yielded 585 results. We focused on the past 10 years (2016-2026), thereby excluding 114 studies. A total of 471 studies remained. Of the 471 articles reviewed, 239 studies were excluded based on their titles, abstracts, publication types, and topics because of inappropriate study designs (i.e., designs other than cross-sectional or animal studies). A total of 232 studies were further investigated. In this review, the analysis focused exclusively on animal studies, and the full texts were assessed against predefined eligibility criteria, including English language, study design, animal model, periodontal exposure, and AD-related outcomes. Studies that met all inclusion criteria were included, whereas articles with irrelevant outcomes were excluded. The main reviewers were NS, CC, and FF, under the supervision of VZ. After full-text screening, 101 studies remained (Table 1).

**Table 1:** Summary of the included studies Aβ, amyloid beta; AD, Alzheimer’s disease; APoE, apolipoprotein E; BBB, blood-brain barrier; BDNF, brain-derived neurotrophic factor; EV, extracellular vesicle; LPS, lipopolysaccharides; MAPK, mitogen-activated protein kinase; MDSC, myeloid-derived suppressor cell; OMVs, outer membrane vesicles; PD, periodontal disease; Pg, *Porphyromonas gingivalis*; RAGE, receptor for advanced glycation end products; ROS, reactive oxygen species; T2D, type 2 diabetes; Tg, transgenic; TNF-α, tumor necrosis factor-alpha

Reference	Author (year)	Study title	Study type	Population/sample	Country	Research setting/method	Main findings	Conclusion
[1]	Wang et al. (2023)	IL-1β/TNF-α role	Animal	AD mice	China	Cytokine model	Neuroinflammation increase	Cytokines link disease
[2]	Furutama et al. (2020)	IL-6 BBB disruption	Animal	Mice	Japan	Cytokine model	BBB damage	Inflammation key
[3]	Ilievski et al. (2018)	Chronic oral application of a periodontal pathogen results in brain inflammation	In vivo animal study	C57BL/6 mice	USA/Australia	Chronic oral infection with Pg	Brain inflammation, Aβ accumulation, neurodegeneration	Oral Pg infection induces Alzheimer-like pathology
[4]	Xue et al. (2020)	Chronic periodontitis induces microbiota-gut-brain axis disorders	Experimental animal study	Mice	China	Gut microbiota sequencing + neuroinflammation analysis	Periodontitis causes gut dysbiosis and brain inflammation	Microbiota-gut-brain axis mediates periodontal effects on the brain
[5]	Liu et al. (2025)	Pg and T2D cognition	Animal	Mice	China	Metabolic model	Cognitive decline	Gut link
[6]	Li et al. (2024)	Gingipains may be one of the key virulence factors of Pg to impair cognition and enhance BBB permeability: an animal study	Experimental animal study	Mice	Multicountry (Journal of Clinical Periodontology affiliation-based collaboration)	Pg gingipain mouse model	Gingipains increase BBB permeability and impair cognition	Gingipains are key virulence factors driving neurodegeneration and BBB disruption
[7]	Qiu et al. (2025)	OMVs vs bacteria	Animal	Mice	China	Injection model	Distinct mechanisms	Both contribute
[8]	Zhang et al. (2018)	Insulin resistance	Animal	Mice	China	Infection model	Aβ/tau increase	Metabolic mechanism
[9]	Lei et al. (2023)	BBB permeability	Animal + in vitro	Mice + cells	China	Bacteremia model	BBB leakage increase	Brain invasion possible
[10]	Jiang et al. (2025)	BBB disruption	Animal	Mice	China	Infection model	Neuronal loss	BBB critical
[11]	Qian et al. (2021)	Periodontitis deteriorates cognitive function and impairs neurons and glia	Experimental animal study	AD/rodent model	China	Cognitive testing + histology	Amyloid increase, neuronal damage, glial dysfunction	Periodontitis accelerates neurodegeneration and cognitive decline
[12]	Ishida et al. (2017)	Pg infection	Animal	Tg mice	Japan	Oral infection	Aβ increase	Causal support
[13]	Jimenez-Harrison (2025)	Ligature AD model	Animal	Tg mice	USA	Ligature model	Amyloid increase	Disease worsens
[14]	Dominy et al. (2019)	Pg in AD brains	Animal + human	Mice + human brains	USA/Poland	Infection + gingipain inhibition	Aβ increase, neuroinflammation reduced by inhibitors	Strong causal link
[15]	Lu et al. (2022)	Salivary microbiota and AD	Animal	30 mice	China	Microbiota transfer	Worse cognition, Aβ increase	Gut-brain axis involved
[16]	Ma et al. (2023)	PG EVs	Animal	Mice	Korea	EV exposure	TNF-α increase, memory loss	EVs drive neurodegeneration
[17]	Qian et al. (2024)	Gut homeostasis disruption	Animal	APP/PS1 mice	China	Ligature + microbiome	Dysbiosis, BBB damage	Gut axis critical
[18]	Xie et al. (2025)	Microglial Aβ clearance	Animal + in vitro	Mice + cells	China	Infection + assay	Reduced Aβ clearance	Immune dysfunction key
[19]	Gong et al. (2022)	OMVs and tau pathology	Animal	Mice	China	OMV injection	Tau phosphorylation increase	OMVs induce AD damage
[20]	Kong et al. (2025)	IFITM3-Aβ axis	Animal	Mice	China	Infection model	IFITM3 and Aβ increase	Immune pathway involved
[21]	Jiang et al. (2021)	GSK3β pathway	Animal + in vitro	APP KI mice	China/Japan	LPS exposure	Tau phosphorylation increase	Key signaling pathway
[22]	Zhu et al. (2025)	Gut-brain axis disruption	Animal	Mice	China	Microbiome analysis	Dysbiosis + inflammation	Oral microbes affect brain
[23]	Leira et al. (2019)	Serum Aβ levels	Animal	Rats	Spain	LPS injection	Aβ increase	Periodontitis increases Aβ
[24]	Nie et al. (2019)	Macrophage Aβ production	Animal + in vitro	Mice + macrophages	China/UK	Infection assay	Aβ increase in immune cells	Peripheral contribution
[25]	Yamawaki et al. (2022)	Imipramine neuroprotection	Animal + in vitro	Mice	Japan	Drug treatment	Inflammation decrease	Protective potential
[26]	Hu et al. (2021)	STAT3 signaling	Animal	Rats	China	Ligature model	Cognitive decline increase	STAT3 pathway involved
[27]	Elashiry et al. (2024)	EVs cross the BBB	Animal + in vitro	Mice + BBB model	USA	BBB tracking	EVs enter brain	Direct brain access
[28]	Hao et al. (2022)	Microglial activation	Animal	APP mice	USA	Infection model	Aβ increase, synapse loss	AD worsens
[29]	Xie et al. (2024)	Neuroinflammation	Animal	Rats	China	Ligature model	Brain inflammation increase	Oral infection harmful
[30]	Magnusson et al. (2024)	NOX4 oxidative stress	In vitro	Microglia	Sweden	Cell assay	ROS increase	Oxidative damage
[31]	Yoshida et al. (2023)	OMVs cognition	Animal	Mice	China	OMV exposure	Memory loss	OMVs neurotoxic
[32]	Zhao et al. (2023)	Nisin therapy	Animal	Mice	USA/Japan	Treatment model	Aβ decrease	Therapeutic effect
[33]	Almarhoumi et al. (2023)	Microglial response	Animal	Mice	USA	Ligature model	Immune activation	Brain inflammation
[34]	Kazemi et al. (2024)	Lactobacilli protection	Animal	Rats	Iran	Probiotic model	Memory improvement	Gut therapy useful
[35]	Cheng et al. (2023)	MDSC therapy	Animal	Mice	China	Cell therapy	Plaques reduction	Immune modulation
[36]	Ciccotosto et al. (2024)	Oral Pg infection	Animal	Mice	USA/Australia	Oral infection	Brain damage	Causal link
[37]	Shen et al. (2024)	APP processing	Animal	Mice	China	Molecular assay	Aβ increase	Peripheral contribution
[38]	Bahar et al. (2021)	Metabolic AD model	Animal	Mice	UK	Metabolic model	Inflammation increase	Comorbidity effect
[39]	Duan et al. (2022)	Insulin signaling	Animal	Rats	China	STZ model	Cognitive decline	Metabolic link
[40]	He et al. (2022)	*Akkermansia*	Animal	Rats	China	Probiotic model	Protection increase	Microbiome protective
[41]	Chen et al. (2025)	*Prevotella* effects	Animal	Mice	China	Infection model	Inflammation increase	Other pathogens matter
[42]	Takeda et al. (2016)	Tooth loss and BDNF	Animal	Mice	Japan	Tooth loss model	Memory decrease	Mastication important
[43]	Katano et al. (2020)	Early tooth loss	Animal	Mice	Japan	Development model	Hippocampal changes	Long-term effects
[44]	Hatakeyama et al. (2025)	Tooth loss decline	Animal	Mice	Japan	Tooth loss model	Decline independent diet	Direct effect
[45]	Singhrao et al. (2025)	APoE + Pg	Animal	APoE mice	UK	Brain infection	Gene activation	Genetic risk
[46]	Luo et al. (2019)	Cerebral blood flow	Animal	Rats	China	Extraction model	Flow decrease	Vascular link
[47]	Oue et al. (2016)	Aging AD interaction	Animal	Tg mice	Japan	Tooth loss model	Pathology increase	Aging worsens
[48]	Taslima et al. (2021)	Gliosis	Animal	Mice	Japan	APP model	Gliosis increase	Neurodegeneration
[49]	Goto et al. (2020)	Trigeminal neurons	Animal	Mice	Japan	Tooth loss model	Neuronal loss	Sensory loss important
[50]	Yavuz et al. (2025)	Treatment effect	Animal	Rats	Turkey	Therapy model	AD pathology decrease	Treatment helps
[51]	Zhao et al. (2025)	Pregnancy Pg exposure	Animal	Mice	China	Prenatal model	Development damage	Systemic risk
[52]	Zhang et al. (2025)	NLRP3 pathway	Animal	Mice	China	Inflammation model	Neuroinflammation increase	Immune activation
[53]	Zeng et al. (2021)	Cofilin-2 link	Animal	Mice	China	Molecular study	Inflammation link	Protein mediator
[54]	Yan et al. (2022)	*Fusobacterium nucleatum* and neurodegeneration	Animal	Rats	China	Infection model	Neurodegeneration + dysbiosis	Broadens pathogen scope
[55]	Wu et al. (2017)	Cathepsin B in AD	Animal	Mice	China	Infection model	AD-like pathology increase	Protease involvement
[56]	Wu et al. (2022)	*Treponema denticola* apoptosis	Animal	Mice	China	Infection model	Neuronal apoptosis increase	Multi-pathogen contribution
[57]	Widziolek (2026)	Zebrafish neuroinflammation	Animal	Zebrafish	Poland	Gingipain exposure	Neuroinflammation increase	Basic model validation
[58]	Wang et al. (2024)	Occlusal support loss	Animal	Rats	China	Tooth loss model	Cognitive decline	Mastication essential
[59]	Tsukahara (2020)	Butyric acid effects	Animal	Mice	Japan	Chemical exposure	Behavioral impairment	Bacterial metabolites toxic
[60]	Tang et al. (2021)	Tau hyperphosphorylation	Animal	Rats	China	Pg infection model	Tau pathology increase	AD hallmark triggered
[61]	Tang et al. (2022)	*T. denticola* hippocampus	Animal	Mice	China	Infection model	Inflammation increase	Brain damage confirmed
[62]	Sun et al. (2024)	NLRP3 pathway	Animal	SAMP8 mice	China	Tooth loss model	Cognitive impairment	Inflammasome role
[63]	Su et al. (2021)	Aβ accumulation	Animal	Mice	China	Infection model	Aβ increase hippocampus	Oral pathogens key
[64]	Shen et al. (2025)	Transcriptomics inflammation	Animal	Mice	China	Single-cell RNA-seq	Endothelial inflammation	Vascular involvement
[65]	Ribas et al. (2020)	Oxidative stress	Animal	Rats	Brazil	Infection model	ROS increase	Redox imbalance
[66]	Oue et al. (2024)	Obesity + Pg	Animal	Mice	Japan	Metabolic model	Cognitive decline increase	Comorbidity effect
[67]	Meng et al. (2025)	Mitochondrial dysfunction	Animal	Rats	China	Tooth loss model	Energy failure	CNS metabolism affected
[68]	Ma et al. (2023a)	Lactobacillus protection	Animal	Mice	China	Probiotic model	Neuroprotection increase	Gut therapy beneficial
[69]	Liu et al. (2025)	Nanocluster therapy	Animal	Mice	China	Drug delivery	Cognitive improvement	Novel therapy promising
[70]	Kim et al. (2023)	GV1001 inhibitor	Animal	Mice	Korea	Ligature model	Inflammation decrease	Therapeutic potential
[71]	Jin et al. (2023)	P38 MAPK pathway	Animal	Rats	China	Infection model	Inflammation increase	Signaling pathway
[72]	Jiang et al. (2024)	Meninges changes	Animal	Mice	China	Transcriptomics	Immune remodeling	Barrier involvement
[73]	Jiang et al. (2025)	SAPK/JNK signaling	Animal	Mice	China	Infection model	Synaptic dysfunction	Stress pathway
[74]	Jiang et al. (2021)	PorX/PorY loop	In vitro	Bacteria	China	Genetic analysis	Virulence regulation	Mechanistic insight
[75]	Huang et al. (2021)	Leptomeningeal IL-1β	Animal	Mice	China	Infection model	Synaptic failure	Cytokine damage
[76]	He et al. (2022)	Adenosine A2A receptor	Animal	Mice	China	Drug model	Neuroprotection increase	Targetable receptor
[77]	Gui et al. (2025)	Synaptic defects	Animal	Mice	China	LPS model	Synaptic loss increase	GSK3β involvement
[78]	Furukawa et al. (2024)	Behavioral symptoms	Animal	APP KI mice	Japan	Tooth loss model	BPSD-like symptoms	Behavioral link
[79]	Furukawa et al. (2023)	Capsaicin effects	Animal	Mice	Japan	Diet intervention	Astrogliosis decrease	Diet modulation
[80]	Elsayed et al. (2023)	Exosomes aging	Animal	Mice	USA	Exosome tracking	Immune aging increase	Vesicle role
[81]	Eid et al. (2025)	Chronic LPS exposure	Animal	Mice	USA	Systemic inflammation	AD pathology increase	Inflammation alone harmful
[82]	Ding et al. (2018)	Memory impairment	Animal	Mice	China	Infection model	Cognitive decline	Age vulnerability
[83]	Díaz-Zúñiga (2020)	Serotype variation	Animal	Rats	Chile	Infection model	Pathology variation	Strain matters
[84]	Dewey and Rishniw (2021)	Dog cognition	Animal	Dogs	USA	Observational	Cognitive decline	Translational model
[85]	de Castro et al. (2024)	Anxiety effects	Animal	Rats	Brazil	Infection model	Anxiety increase	Psychological impact
[86]	Chen et al. (2024)	GV1001 protection	Animal	Mice	Korea	Drug model	Systemic protection	Therapeutic effect
[87]	Chen et al. (2025)	Herbal therapy	Animal	Rats	China	Gut-brain treatment	Recovery increase	Traditional medicine
[88]	Chen et al. (2023)	Sex differences	Animal	Mice	China	Infection model	Cognitive variation	Demographic factors
[89]	Cafferata et al. (2024)	Peri-implantitis	Animal	Rats	Chile	Implant infection	Brain dysfunction	Dental implants risk
[90]	Aravindraja (2022)	Brain infection model	Animal	Mice	USA	Direct infection	Aβ increase	Strong causation
[91]	Aravindraja (2023)	miRomics analysis	Animal	Mice	USA	Omics study	Molecular changes	Pathogen footprint
[92]	Han et al. (2019)	Extracellular RNA	Animal	Mice	Korea	Vesicle tracking	BBB inflammation	Genetic material effect
[93]	Zhou et al. (2025)	Th17 pathway	Animal	Mice	China	Infection model	Microglial activation	Immune cascade
[94]	Kantarci et al. (2020)	Microglial response	Animal	Mice	USA	Infection model	Immune activation	Brain response confirmed
[95]	Arastu-Kapur (2020)	COR388 inhibitor	Animal	Dogs	USA	Drug trial	Pathology reduction	Therapeutic candidate
[96]	Chi et al. (2021)	Glymphatic dysfunction	Animal	Mice	China	Dye tracking	Waste clearance decrease	Clearance failure
[97]	Liu et al. (2020)	Salvianolic acid B	Animal	Mice	China	Drug intervention	Aβ decrease	Protective compound
[98]	Gu et al. (2020)	Bone loss correlation	Animal	Mice	China	Correlation study	AD pathology increase	Bone loss marker
[99]	Zeng et al. (2021)	RAGE endothelial pathway	Animal + in vitro	Mice	China	Endothelial model	Aβ accumulation increase	Vascular mechanism
[100]	Zhang et al. (2021)	Th17/Treg imbalance via STAT3 activation modulates cognitive impairment	Experimental immunology study	Rodent model	China	STAT3 signaling + immune profiling	Immune imbalance leads to neuroinflammation and cognitive decline	Adaptive immune dysregulation links PD to AD
[101]	Wu et al. (2022)	The Periodontal Pathogen *Fusobacterium nucleatum* Exacerbates Alzheimer's Pathogenesis via Specific Pathways	Experimental animal study	Mice	China/USA (multi-institutional collaboration)	AD mouse model with *F. nucleatum* infection	*F. nucleatum* increases amyloid deposition and neuroinflammation	Periodontal pathogens contribute to AD progression beyond PG

Data from the included studies were extracted and synthesized qualitatively. The analysis focused on identifying common mechanistic pathways and patterns across studies (Table 2, Table 3, Table 4, Table 5, Table 6, Table 7).

**Table 2:** BBB and systemic inflammation (n = 7) 3xTg-AD, triple-transgenic Alzheimer’s disease; Aβ, amyloid beta; AD, Alzheimer’s disease; ApoE, apolipoprotein E; BBB, blood-brain barrier; BMECs, brain microvascular endothelial cells; LPS, lipopolysaccharides; PD, periodontal disease; Pg, Porphyromonas gingivalis; TNF-α, tumor necrosis factor-alpha

No.	Author (year)	Species/strain	AD/disease model	Periodontal induction/exposure	Duration	Primary outcomes	Key findings
1	Wang et al. (2023) [1]	Female 3xTg-AD mice and B6129SF2/J non-transgenic mice	3xTg-AD transgenic mouse model of AD	Heat-killed PG injection, ligature-induced periodontitis, and local IL-1beta/TNF-alpha injections	5 weeks	IL-1beta/TNF-alpha expression, neuroinflammation, glial activation, tau phosphorylation, Abeta accumulation, cognitive impairment	Periodontitis-induced systemic inflammation and worsened cognitive impairment
2	Furutama et al. (2020) [2]	Female C57BL/6J mice	Ligature-induced neuroinflammation/BBB disruption model	Ligature-induced periodontitis around the second maxillary molars	1-8 weeks; main hippocampal analysis at 1 week	Serum IL-6, hippocampal IL-1beta, claudin-5, BBB permeability, dextran leakage	PD-induced neuroinflammation and BBB disruption linked to cognitive impairment risk
3	Li et al. (2024) [6]	Male C57BL/6 mice	Cognitive impairment/BBB dysfunction model	Tail-vein injection of Pg, gingipains, or Pg-LPS three times weekly	8 weeks	Cognition, BBB permeability, hippocampal/cortical pathology, tau tangles, Mfsd2a/Cav-1 signaling, Ddx3x regulation	Pg and gingipains impaired learning and memory, induced neuronal degeneration
4	Lei et al. (2023) [9]	Sprague-Dawley rats; bEnd.3 BMECs and U87 astrocyte BBB co-culture	Pg bacteremia/BBB dysfunction model	Tail-vein Pg injection; in vitro BBB exposure	8 weeks in vivo; 24 hours in vitro	Evans blue infiltration, albumin deposition, BBB permeability, Cav-1 and Mfsd2a, transcytosis	Pg bacteremia increased BBB permeability and promoted bacterial translocation into brain tissue
5	Jiang et al. (2025) [10]	Male C57BL/6 mice; lymphatic endothelial cells	Pg-induced periodontitis with cognitive impairment model	Oral topical application of Pg W83 to the gingiva and buccal mucosa	8 weeks in vivo; 24 hours in vitro	Cognition, neuroinflammation, tau hyperphosphorylation, neuronal loss, BBB permeability, meningeal lymphatic drainage	Pg-induced periodontitis promoted neuroinflammation
6	Kim et al. (2023) [70]	C57BL/6 and ApoE-deficient mice	Periodontitis-associated atherosclerosis	Silk ligatures	6 weeks	Bone loss, vascular inflammation	GV1001 reduced periodontal destruction and vascular inflammation
7	Jiang et al. (2024) [72]	C57BL/6 mice	Pg-induced cognitive impairment	Chronic oral Pg W83	16 weeks	Barrier dysfunction, transcriptomics	Meningeal immune dysregulation contributed to cognitive impairment

**Table 3:** Gut-brain axis and microbiome dysbiosis (n = 7) AD, Alzheimer’s disease; BBB, blood-brain barrier; BDNF, brain-derived neurotrophic factor; CFU, colony-forming units; LPS, lipopolysaccharide; pEV, periodontal pathogen-derived extracellular vesicles; SCFA, short-chain fatty acids; TNF-α, tumor necrosis factor-alpha

No.	Author (year)	Species/strain	AD/disease model	Periodontal induction/exposure	Duration	Primary outcomes	Key findings
1	Xue et al. (2020) [4]	C57BL/6J mice	No transgenic AD	Long-term ligature-induced chronic periodontitis around the left maxillary second model molar	12 months	Cognitive impairment, microbiota dysbiosis, intestinal barrier disruption, BBB impairment, neuroinflammation, TLR4/NF-kB activation	Chronic periodontitis caused progressive cognitive decline, oral and gut microbiota dysbiosis, increased systemic inflammation
2	Liu et al. (2025) [5]	Male C57BL/6J mice	T2DM model induced by high-fat diet + STZ	Intravenous administration of PG, 1 x 10^8 CFU, 3 times/week	4 weeks Pg exposure after diabetes induction	Cognitive impairment, hippocampal neuron injury, synaptic plasticity, microglial inflammation, gut dysbiosis, SCFA metabolism, Olfr78	Pg aggravated cognitive dysfunction by reducing synaptic plasticity, enhancing neuroinflammation, disrupting gut microbiota
3	Lu et al. (2022) [15]	APPswe/PS1dE9 mice; human saliva samples	Transgenic AD mouse model	Oral gavage with periodontitis-related salivary microbiota versus healthy salivary microbiota	2 months	Cognition, anxiety-like behavior, Abeta, neuroinflammation, gut dysbiosis, intestinal inflammation/barrier, systemic inflammation	Periodontitis-related salivary microbiota aggravated AD
4	Ma et al. (2023) [16]	Male C57BL/6 mice	Pg/pEV-induced cognitive impairment model	Gingival exposure or oral gavage of Pg or Pg-derived extracellular vesicles	21 days exposure; 5 days FITC-pEV tracing	Memory impairment, neuroinflammation, BDNF/NMDAR, blood LPS/TNF-alpha, gut dysbiosis, trigeminal pEV translocation	pEVs were detected in the trigeminal ganglia and hippocampus, suggesting trigeminal nerve-mediated transport
5	Qian et al. (2024) [17]	3-month-old male APP/PS1 mice	APP/PS1 AD mouse model	Bilateral silk ligation of maxillary second molars + local Pg-LPS injections	8 weeks	Cognition, anxiety-like behavior, Abeta, neuroinflammation, gut dysbiosis, intestinal/BBB dysfunction, synaptic injury	Periodontitis worsened novel object recognition, and open-field outcomes
6	Zhu et al. (2025) [22]	C57BL/6 mice	AD-like neurodegeneration model	Oral Pg W83 infection	3 and 6 months	Gut dysbiosis, kynurenine pathway imbalance, 3-HK, neuronal apoptosis, cognition	Pg induced oral-gut microbiota dysbiosis and disrupted tryptophan/kynurenine metabolism
7	Yan et al. (2022) [54]	Male Sprague-Dawley rats	D-galactose + AlCl3 AD-like rat model	Ligatures around maxillary second molars + oral *Fusobacterium nucleatum*	6 weeks within 22-week AD induction	Cognition, bone loss, Abeta1-42, pTau181, serum LPS, gut microbiota	*F. nucleatum* increased alveolar bone loss, worsened MWM cognition, enhanced neuronal degeneration/vacuolation

**Table 4:** Behavior/cognition (n = 6) AD, Alzheimer’s disease; BA-LPS, butyric acid-lipopolysaccharide complex; BDNF, brain-derived neurotrophic factor; CFU, colony-forming units; GSH, glutathione; LPS, lipopolysaccharide; MWM, Morris water maze; Pg, *Porphyromonas gingivalis*; pEV, periodontal pathogen-derived extracellular vesicles; SCFA, short-chain fatty acids; TBARS, thiobarbituric acid reactive substances; TNF-α, tumor necrosis factor-alpha; TrkB, tropomyosin receptor kinase B; TS, total sulfhydryl

No.	Author (year)	Species/strain	AD/disease model	Periodontal induction/exposure	Duration	Primary outcomes	Key findings
1	Wang et al. (2024) [58]	Male Sprague-Dawley rats, young and middle-aged	Occlusal support loss/tooth extraction model	Bilateral extraction of all maxillary molars	Young, 37 days; middle-aged, 72 days before	Spatial cognition, BDNF/AKT pathway, PSD95, NMDAR, trigeminal-hippocampal morphology	Occlusal support loss impaired MWM spatial cognition.
2	Tsukahara et al. (2020) [59]	Male ICR mice, 14 weeks	Periodontitis-associated metabolite exposure model	Consecutive intra-gingival injections of Pg metabolites: LPS, n-butyric acid, or BA-LPS	32 days	Open-field behavior, hippocampal IL-6/BDNF/TrkB, cytokines, SCFAs, neurotransmitters, histopathology	Periodontal metabolites can alter behavior and neuroimmune signaling.
3	Ribas et al. (2020) [65]	Male Wistar rats	Scopolamine-induced cognitive deficit model	Ligature-induced PD combined with scopolamine	1 month total; final evaluation 14 days after ligature	Bone loss, oxidative stress markers GSH/TS/catalase/superoxide/TBARS, spatial memory, inhibitory avoidance	PD+scopolamine groups had greater alveolar bone loss than scopolamine alone.
4	Meng et al. (2025) [67]	Male Wistar rats	Tooth loss, cognitive impairment	Bilateral maxillary molar extraction	8 weeks	MWM cognition, mitophagy markers	Tooth loss caused mitochondrial dysfunction and impaired mitophagy linked to cognitive decline.
5	Ma et al. (2023) [68]	Male C57BL/6 mice	Pg-induced cognitive impairment	Gingival exposure to PG or pEVs	21 days	Neuroinflammation, gut dysbiosis	Periodontitis induced cognitive impairment via microbiota-gut-brain mechanisms.
6	Dewey and Rishniw (2021) [84]	Aging dogs	Canine cognitive dysfunction	Natural PD	Cross-sectional	Cognitive scores	More severe periodontitis correlated with worse cognition.

**Table 5:** Aβ and tau pathology (n = 19) Aβ, amyloid beta; AD, Alzheimer’s disease; ApoE, apolipoprotein E; BBB, blood-brain barrier; CatB, cathepsin B; CFU, colony-forming units; iNOS, inducible nitric oxide synthase; LPS, lipopolysaccharide; MWM, Morris water maze; Pg, *Porphyromonas gingivalis*; RAGE, receptor for advanced glycation end-products; STZ, streptozotocin; TGFBR1, transforming growth factor beta receptor 1; TNF-α, tumor necrosis factor-alpha; Vmes, mesencephalic trigeminal nucleus

No.	Author (year)	Species/strain	AD/disease model	Periodontal induction/exposure	Duration	Primary outcomes	Key findings
1	Ilievski et al. (2018) [3]	C57BL/6 wild-type mice	Non-transgenic WT mouse model	Chronic oral application of PG with gingipain	22 weeks	Neuroinflammation, neurodegeneration, Abeta42 production, tau phosphorylation, microgliosis, astrogliosis	Chronic oral Pg infection induced AD-like pathology, including brain inflammation
2	Qian et al. (2021) [11]	APP/PS1 transgenic male mice	APP/PS1 AD transgenic model	Pg-LPS gingival injections + ligature-induced periodontitis	8 weeks	Cognitive impairment, Abeta40/42 increase, neuronal loss, glial activation, inflammatory cytokines	Periodontitis worsened learning and memory, increased Aβ, and neuroinflammation
3	Leira et al. (2019) [23]	Male Sprague-Dawley rats	Experimental AD-related amyloid response model	Pg-LPS injections into the palatal gingiva	14 days induction + 21 days follow-up	Alveolar bone loss, serum Abeta1-40 and Abeta1-42	Pg-LPS-induced periodontitis increased periodontal inflammation and systemic amyloid burden.
4	Nie et al. (2019) [24]	12-month-old female C57BL/6J mice; RAW264.7 macrophages; human gingiva	Peripheral amyloidogenesis/AD-related inflammatory model	Chronic systemic Pg infection via intraperitoneal injection	Every 3 days for 3 weeks	Liver macrophage inflammation, APP770, CatB, Abeta1-42, Abeta3-42, NF-kB, macrophage viability/phagocytosis	CatB/NF-kB inhibition reduced inflammatory/amyloidogenic responses.
5	Kazemi et al. (2024) [34]	Male Wistar rats	Pg-induced AD-like pathology	Two palatal Pg ATCC33277 injections; oral lactobacilli mixture prevention/treatment	8 weeks of oral gavage after injections	Gingival inflammation, MWM, passive avoidance, hippocampal Abeta plaques, trace elements	Pg induced gingival inflammation, MWM and passive avoidance deficits
6	Shen et al. (2024) [37]	C57BL/6J mice	WT model evaluating AD-related changes	Oral live Pg ATCC33277 after oral flora suppression	8 weeks	Bone resorption, cognition, bacterial dissemination, cytokines, APP isoforms, BACE1, Abeta1-40/42	Pg-induced periodontitis, reduced bone mineral density, disseminated to the gingiva/blood/cortex, impaired MWM and passive avoidance performance
7	He et al. (2022) [40]	Adult male Sprague-Dawley rats	D-galactose + AlCl3-induced AD-like rat model	Ligature-induced PD; Akkermansia muciniphila applied orally to ligatures	14 weeks AD induction; 6 weeks PD/probiotic	Cognition, bone loss, Abeta, metabolic markers, gut microbiota, gut-brain axis	A. muciniphila improved learning, reduced alveolar bone destruction, and attenuated neuronal degeneration
8	Singhrao and Consoli (2025) [45]	Male ApoE-/- mice	ApoE knockout AD susceptibility model	Oral mono-infection with Pg FDC381, confirmed brain entry by PCR	12 and 24 weeks post-infection	Expression of 92 AD-associated genes	Pg brain invasion altered AD-related gene expression
9	Oue et al. (2016) [47]	Female Tg2576 APP transgenic mice	Tg2576 AD model with amyloid-beta deposition	Bilateral maxillary molar extraction at 14 months	4 months; evaluated at 18 months	Passive avoidance, Abeta40/42, hippocampal Abeta deposition, CA1/CA3 pyramidal cells	Tooth loss after advanced AD onset may not substantially aggravate amyloid pathology or memory impairment.
10	Goto et al. (2020) [49]	Female 3xTg-AD mice	Triple-transgenic AD model APPswe/PS1M146V/tauP301L	Bilateral extraction of maxillary molars	1 week to 4 months	Abeta42 in Vmes, Vmes neuronal loss, LC degeneration, hippocampal neuronal loss, microglia, Barnes maze	Tooth extraction injured Vmes neurons containing cytotoxic intracellular Aβ.
11	Yavuz et al. (2025) [50]	Male Sprague-Dawley rats	STZ-induced AD-like neurodegeneration model	Ligature-induced PD; nonsurgical periodontal therapy with 2% chlorhexidine	21-41 days	Passive avoidance, MWM, p-tau/tau, NLRP3, BACE1, IL-1beta, iNOS, NF-kB, bone loss	Periodontal treatment reduced NF-kB in AD+PD, suggesting neuroinflammation attenuation.
12	Zhao et al. (2025) [51]	Pregnant mice and embryos	No AD model	Intravenous and oral sonicated Pg during pregnancy	Embryonic day 2.5-16.5	Cleft palate, osteogenesis, efferocytosis, glycolysis, H4K12la, ADAM17, TGFBR1, MerTK	Maternal Pg exposure caused cleft palate in offspring, impaired palatal fusion, and osteogenesis
13	Zhang et al. (2025) [52]	Female C57BL/6J mice, 12-13 months	Middle-aged sporadic AD-like model	Ligature-induced PD + oral Pg every other day	24 weeks	Cognition, Abeta42/fragments, tau, neuroinflammation, NLRP3, gut dysbiosis, intestinal/BBB integrity, synaptic/neuronal markers	Chronic Pg periodontitis impaired cognition/exploration, caused bone loss and Pg brain detection
14	Zeng et al. (2021) [53]	APP/PS1 mice and SK-N-SH APPwt cells	APP/PS1 AD model	Pg-LPS palatal injections + ligatures around maxillary molars	3 months in vivo; 72 h-7 days in vitro	Cognition, proteomics, inflammation proteins, tau, PP2A, Cofilin 2	Chronic PD impaired MWM performance
15	Tang et al. (2021) [60]	Male Sprague-Dawley rats; HT-22 cells	WT rat neuroinflammation/tauopathy model	Intravenous tail-vein Pg ATCC33277, 10^8 CFU/200 uL, 3 times/week	4 or 12 weeks	pTau181/pTau231, hippocampal cytokines, GFAP, systemic inflammation, PP2A, GSK-3beta	Peripheral Pg infection increased hippocampal tau phosphorylation
16	Tang et al. (2022) [61]	Male C57BL/6 mice; BV2 microglia; N2a cells	WT mouse tauopathy/neuroinflammation model	Oral *Treponema denticola*; Pg and mixed infection compared	24 weeks, 3 oral infections/week	Tau Thr181/Thr231/Ser396, neuroinflammation, microglia, GSK3beta, systemic inflammation, bone loss, brain colonization	T. denticola caused periodontitis, colonized the hippocampus, and carotid arteries
17	Su et al. (2021) [63]	Male C57BL/6 mice	WT model evaluating AD-like amyloid pathology	Oral *Treponema denticola*; Pg positive control	24 weeks, 3 times/week	Hippocampal Abeta1-40/1-42, bacterial dissemination, BACE1/PS1/PS2	T. denticola disseminated to the hippocampus, aorta, and trigeminal ganglion
18	Liu et al. (2020) [97]	12-month-old mice	Pg-induced AD-like impairment	Intraperitoneal Pg injection	3-4 weeks	Aβ metabolism	Salvianolic Acid B improved cognition.
19	Zeng et al. (2021) [99]	Female mice + endothelial cells	Cerebrovascular amyloidogenesis	Systemic Pg infection	3 weeks	RAGE, Aβ influx	Pg enhanced BBB amyloid- β transport.

**Table 6:** Bacterial dissemination and microbial mechanisms (n = 2) AD, Alzheimer’s disease; Pg, *Porphyromonas gingivalis*; TNF-α, tumor necrosis factor-alpha

No.	Author (year)	Species/strain	AD/disease model	Periodontal induction/exposure	Duration	Primary outcomes	Key findings
1	Dominy et al. (2019) [14]	BALB/c mice; SH-SY5Y cells; human AD brain tissue and CSF	Human AD tissue analysis and experimental oral infection mouse model	Oral infection with Pg W83; gingipain exposure and inhibition studies	6-10 weeks in mice	Brain colonization, gingipains, Abeta1-42, tau fragmentation, neuroinflammation, neuronal loss	Pg DNA and gingipain antigens were identified in AD brains
2	Jiang et al. (2021) [74]	PG ATCC 33277 and W83 strains; BALB/c mice	Virulence-regulation and mechanistic periodontal pathogenesis model	Genetic analysis of the PorX/PorY two-component system and the σP regulatory pathway in PG	Ιn vitro assays; 30-day mouse virulence observation	PorX/PorY-σP transcriptional regulation, Type IX secretion system (T9SS), gingipain-related virulence, promoter binding, mouse survival	PG virulence in mice, confirming their importance in pathogenicity

**Table 7:** Neuroinflammation and microglial dysfunction (n = 60) Aβ, amyloid beta; AD, Alzheimer’s disease; ApoE, apolipoprotein E; BBB, blood-brain barrier; CatB, cathepsin B; CFU, colony-forming units; EC, endothelial cells; HFD, high-fat diet; LPS, lipopolysaccharide; MAPK, mitogen-activated protein kinase; MCI, mild cognitive impairment; mMDSC, monocytic myeloid-derived suppressor cell; MWM, Morris water maze; OMV, outer membrane vesicle; Pg, *Porphyromonas gingivalis*; TLR4, toll-like receptor 4; TNF-α, tumor necrosis factor-alpha

No.	Author (year)	Species/strain	AD/disease model	Periodontal induction/exposure	Duration	Primary outcomes	Key findings
1	Qiu et al. (2025) [7]	Male C57BL/6J mice	Pg/Pg OMV-induced neuroinflammation and cognitive impairment model	Intravenous injection of PG or Pg OMVs	12 weeks	Cognitive impairment, astrocyte/microglial activation, inflammatory cytokines, NLRP3 inflammasome activation, and BBB integrity	Both Pg and Pg OMVs induced cognitive decline and neuroinflammation.
2	Zhang et al. (2018) [8]	Male C57BL/6 mice	Pg-LPS-induced cognitive dysfunction model	Intraperitoneal Pg-LPS with or without TLR4 inhibitor TAK-242	7 days	Cognitive performance, astrocyte/microglial activation, inflammatory cytokines, TLR4/NF-kB signaling	Pg-LPS impaired learning and memory while increasing microglial activation and inflammatory cytokine expression.
3	Ishida et al. (2017) [12]	Female hAPP-J20 transgenic mice	APP transgenic AD mouse model	Oral inoculation with Pg, 10^9 CFU in carboxymethylcellulose	5 weeks	Cognitive impairment, alveolar bone loss, Abeta40/42, amyloid plaques, TNF-alpha, IL-1beta, endotoxin	Pg-induced periodontitis significantly exacerbated AD pathology by increasing amyloid deposition.
4	Jimenez-Harrison et al. (2025) [13]	Female 3xTg-AD and non-transgenic mice	3xTg-AD familial AD mouse model	Ligature-induced periodontitis using a unilateral silk ligature around the maxillary second molar	7 or 14 days	Alveolar bone loss, local and neuroinflammation, cognition, synaptic dysfunction, Abeta deposition, and neurodegeneration	Ligature-induced periodontitis caused alveolar bone loss and exaggerated inflammatory responses, hippocampal-dependent memory impairment.
5	Xie et al. (2025) [18]	Male APP/PS1 mice; primary microglia	APP/PS1 AD mouse model	Oral gavage with Pg strains ATCC33277, W50, W83; gingipain-deficient KDP136 and inhibitors	8 weeks	Microglial Abeta phagocytosis/clearance, Abeta deposition, cognition, CD14 signaling, gingipains	Pg strains impaired microglial Abeta internalization and clearance, increased cortical/hippocampal amyloid plaques, and worsened cognition.
6	Gong et al. (2022) [19]	14-month-old male C57BL/6 mice; BV2 and N2a cells	Aging mouse model with AD-like pathology induction	Oral gavage of Pg OMVs; in vitro Pg OMV stimulation	8 weeks in vivo	Memory, BBB integrity, neuroinflammation, tau phosphorylation, NLRP3 inflammasome	Pg OMVs crossed the BBB, impaired spatial memory, increased tau phosphorylation, and activated astrocytes and microglia.
7	Kong et al. (2025) [20]	Male C57BL/6J and APP/PS1 mice; BV2, C6, neuron-glial cultures	APP/PS1 AD and WT AD-like pathology models	Ligature-induced PD + repeated Pg W83 infection	16 weeks	Cognition, BBB permeability, glial activation, IFN-beta/IFITM3, Abeta, neuroinflammation	Pg-induced periodontitis caused bone loss, impaired memory, increased BBB permeability, and activated astrocytes/microglia.
8	Jiang et al. (2021) [21]	APPNL-F/NL-F mice; MG6 microglia and N2a neurons	APP knock-in AD model	Daily intraperitoneal PgLPS, 1 mg/kg	3 weeks	Learning/memory, tau hyperphosphorylation, neuroinflammation, GSK3beta, cytokines	PgLPS impaired memory performance and increased tau phosphorylation.
9	Yamawaki et al. (2022) [25]	C57BL/6J mice; MG-6 microglia; Neuro-2A neurons	Neuroinflammation/AD-related inflammatory model	Intraperitoneal Pg-LPS with/without imipramine	Acute; 2-24 h	Hippocampal inflammation, microglial activation, NF-kB, neuronal viability	Peripheral Pg-LPS induced sustained hippocampal inflammation and microglial activation.
10	Hu et al. (2021) [26]	Male Sprague-Dawley rats	Ligature-induced PD-associated cognitive impairment model	Ligatures around maxillary first molars; pSTAT3 inhibitor cryptotanshinone	8 weeks	MWM cognition, neuroinflammation, STAT3, APP processing, Abeta	PD caused alveolar bone loss, cytokine elevation, glial activation, and memory impairment.
11	Elashiry et al. (2024) [27]	Male C57BL/6 mice; human gingival tissues	BBB dysfunction and neuroinflammatory transport model	Oral Pg-induced periodontitis; PD-derived exosomes isolated	6 weeks Pg; BBB assays 0.5-24 h	BBB permeability, exosome trafficking, and Pg virulence factors	PD-derived exosomes crossed the BBB and accumulated in the hippocampus.
12	Hao et al. (2022) [28]	C57BL/6 WT and AppNL-G-F/NL-G-F mice	APP knock-in AD model	Oral Pg gavage after antibiotic pretreatment	6 weeks of infection	Cognition, Abeta, neuroinflammation, complement, microglia	Pg accelerated cognitive decline and increased Aβ.
13	Xie et al. (2024) [29]	Male C57BL/6 mice	Chronic periodontitis model	Ligature around maxillary second molars	8 weeks	Bone loss, neuroinflammation, cognition	Chronic PD caused hippocampal inflammation and cognitive impairment.
14	Magnusson et al. (2024) [30]	HMC3 microglia and SH-SY5Y neurons	In vitro neuroinflammation model	Pg W50, gingipain mutants, Pg-LPS exposure	6-48 h	ROS, cytokines, tau, neuronal viability	Pg increased oxidative stress and phosphorylated tau.
15	Yoshida et al. (2023) [31]	Female BALB/cAJcl mice; HMC3 microglia	Pg OMV neuroinflammation model	Intraperitoneal Pg OMVs	12 weeks	Tau, cytokines, microglia	Pg translocated to brain regions near ventricles.
16	Zhao et al. (2023) [32]	Female BALB/cByJ mice	Polymicrobial AD-like model	Oral Pg, T. denticola, T. forsythia, F. nucleatum	8 weeks	Brain microbiome, cytokines, Abeta, tau	Pathogen DNA detected in brain tissue.
17	Almarhoumi et al. (2023) [33]	C57BL/6 WT mice; BV2 microglia	Microglial activation model	Ligature-induced periodontitis	1-30 days	Cytokines, microglial markers, Abeta phagocytosis	PD microbiota activated microglia.
18	Cheng et al. (2023) [35]	Female 5xFAD mice	5xFAD AD model	Oral Pg W83 + mMDSCs	4-6 weeks	Cognition, Abeta, neuronal loss	Pg worsened cognition and increased Aβ.
19	Ciccotosto et al. (2024) [36]	Female C57BL/6 mice	WT neurodegeneration model	Oral Pg W50, T. denticola	12 weeks	Bone loss, neurodegeneration, Abeta, tau	Pg increased neuronal damage.
20	Bahar et al. (2021) [38]	Obese diabetic db/db mice	Obesity/type-2 diabetes model	Oral Pg W83 infection	16 weeks	Abeta, tau, neuroinflammation	Pg induced AD-like alterations.
21	Duan et al. (2022) [39]	Sprague-Dawley rats	AD rat model	Ligature + Pg coating + STZ	45 days	Cognition, tau, inflammation	Periodontitis aggravated memory deficits.
22	Chen et al. (2025) [41]	C57BL/6J mice	Pg-associated cognitive impairment model	Ligature + P. nigrescens	6 weeks	Cognition, neuronal degeneration	P. nigrescens worsened memory impairment.
23	Takeda et al. (2016) [42]	C57BL/6J mice	Tooth loss cognitive model	Molar extraction	4-16 weeks	Memory, BDNF	Tooth loss impaired memory retention.
24	Katano et al. (2020) [43]	SAMP8 mice	Aging dementia model	Molar extraction	8 months	Hippocampal ultrastructure	Early tooth loss caused hippocampal remodeling.
25	Hatakeyama et al. (2025) [44]	SAMP8 mice	Aging cognitive decline model	Molar extraction	6 months	Barnes maze, apoptosis markers	Tooth loss impaired learning.
26	Luo et al. (2019) [46]	Wistar rats	Tooth-loss cognitive model	Molar extraction	12 weeks	MWM, blood flow	Tooth loss impaired memory and blood flow.
27	Taslima et al. (2021) [48]	AppNL-G-F mice	AD knock-in model	Molar extraction	2 months	Memory, synapses	Tooth loss impaired memory.
28	Wu et al. (2017) [55]	C57BL/6N mice	LPS-induced AD-like model	Systemic PgLPS	5 weeks	Learning, microglia	Memory deficits via the CatB pathway.
29	Wu et al. (2022) [56]	C57BL/6 mice	Periodontitis model	T. denticola infection	24 weeks	Neuronal apoptosis	Chronic infection induced apoptosis.
30	Widziolek et al. (2026) [57]	Zebrafish larvae	Neuroinflammation model	Pg W83 infection	48 h	Microglia, BBB integrity	Gingipain-null Pg was less inflammatory.
31	Sun et al. (2024) [62]	SAMP8 mice	Aging/AD model	Tooth extraction	2 months	Memory, IL-1β	Tooth loss impaired cognition.
32	Shen et al. (2025) [64]	C57BL/6 mice	Periodontitis cognitive model	Ligature	6 weeks	Endothelial inflammation	Hippocampal EC dysfunction central.
33	Oue et al. (2024) [66]	C57BL/6J mice	Obesity-associated cognitive model	Pg infection + HFD	6 weeks	Cognition, inflammation	Pg worsened the obesity-related decline.
34	Liu et al. (2025) [69]	C57BL/6 mice	Pg-induced AD-like model	Oral Pg infection	6 weeks	Aβ, tau, and inflammasome	Nanoparticles reduced inflammation.
35	Jin et al. (2023) [71]	Sprague-Dawley rats	Periodontitis MCI model	Ligature + Pg W83	10 weeks	APP/BACE1	MAPK pathway involvement.
36	Jiang et al. (2025) [73]	C57BL/6 mice	PgOMV AD model	Pg-derived OMVs	4-8 weeks	Tau phosphorylation	Cathepsin B-mediated effects.
37	Huang et al. (2021) [75]	C57BL/6J mice	Aging neurodegeneration model	Pg infection	3 weeks	NLRP3	IL-1β neuroinflammation.
38	He et al. (2022) [76]	C57BL/6J mice	Cognitive impairment model	PgLPS injection	4 weeks	Glutamate toxicity	Adenosine pathway involvement.
39	Gui et al. (2025) [77]	APPNL-F/NL-F mice	AD model	Systemic PgLPS	7 days	Synaptic proteins	Microglial activation.
40	Furukawa et al. (2024) [78]	AppNL-G-F mice	AD model	Molar extraction	3 months	BDNF, serotonin	Tooth loss worsened dysfunction.
41	Furukawa et al. (2023) [79]	C57BL/6N mice	Aging model	Molar extraction	3 months	TRPV1, BDNF	Capsaicin improved cognition.
42	Elsayed et al. (2023) [80]	C57BL/6 mice	Inflammaging model	Ligature periodontitis	21 days	Immune senescence	Dysbiosis accelerated immune aging.
43	Eid et al. (2025) [81]	C57BL/6J mice	LPS-induced AD model	Pg-LPS infusion	28 days	APP, tau	Peripheral LPS-induced pathology.
44	Ding et al. (2018) [82]	C57BL/6J mice	Age-dependent inflammation	Pg gavage	6 weeks	Cytokines	Middle-aged mice more susceptible.
45	Díaz-Zúñiga et al. (2020) [83]	Sprague-Dawley rats	AD-like periodontal infection	Pg injections	45-55 days	Aβ42, tau	Virulent Pg-induced pathology.
46	de Castro et al. (2024) [85]	Wistar rats	Neurobehavioral impairment	Ligature	14 days	Oxidative stress	Hippocampal dysfunction.
47	Chen et al. (2024) [86]	ApoE-deficient mice	AD-like pathology	Pg inoculation	5 weeks	Atherosclerosis, inflammation	GV1001 reduced pathology.
48	Chen et al. (2025) [87]	Sprague-Dawley rats	Cognitive impairment model	Ligature + Pg	10 weeks	Gut microbiota, BBB	HLJDD improved cognition.
49	Chen et al. (2023) [88]	APP/PS1 mice	AD model	No induction	3-12 months	Periodontal destruction	AD worsened PD
50	Cafferata et al. (2024) [89]	Wistar rats	Neuroinflammation model	Peri-implantitis	28 weeks	Microgliosis	Neuroinflammation observed.
51	Aravindraja et al. (2022) [90]	APP-TgCRND8 mice	AD model	Chronic periodontal infection	12 weeks	Amyloid plaques	Pg increased the amyloid burden.
52	Aravindraja et al. (2023) [91]	C57BL/6J mice	Periodontitis model	T. forsythia infection	8-16 weeks	miRNA profiling	AD-related pathways linked.
53	Han et al. (2019) [92]	U937 cells and mice	Mechanistic inflammation model	Aa OMV injection	Hours	BBB crossing	Bacterial RNA induced TNF-α.
54	Zhou et al. (2025) [93]	Humans and mice	Cognitive impairment model	PgLPS injection	4 weeks	Th17/Treg imbalance	STAT3 involvement.
55	Kantarci et al. (2020) [94]	5xFAD mice	AD model	Ligature periodontitis	4 weeks	Aβ42, microglia	Persistent inflammation.
56	Arastu-Kapur et al. (2020) [95]	Beagle dogs	PD model	Natural infection	28-90 days	Gingipain activity	COR388 reduced activity.
57	Chi et al. (2021) [96]	C57BL/6J mice	AD-like impairment	Pg gavage	4 weeks	Amyloid plaques	Pg increased amyloid accumulation.
58	Gu et al. (2020) [98]	DBA/2 mice	AD pathology model	PgLPS injection	3 weeks	IL-6/IL-17	Memory impairment induced.
59	Zhang et al. (2021) [100]	C57BL/6 mice	Cognitive impairment model	PgLPS injection	4 weeks	STAT3	Inhibition improved cognition.
60	Wu et al. (2022) [101]	5XFAD mice	AD model	Ligature + *Fusobacterium nucleatum*	2 months	Tau phosphorylation	Infection worsened cognition.

One of the shared results of several studies is the role of *P. gingivalis *and its virulence factors in the pathology of AD. *P. gingivalis *infection has been associated with the detection of bacterial DNA and gingipains in brain tissue in animal studies [14,92]. Periodontal pathogens may reach the CNS system and may contribute to neuroinflammatory/neurodegenerative changes in some models, thus contributing to the process of neurodegeneration [9,10]. Preclinical findings have been reported that inhibition of gingipain decreased bacterial load, amyloid-β accumulation, and neurodegeneration, which are key factors in disease development [6,14].

Neuroinflammation has become a key factor linking periodontitis and AD. In some models of periodontitis induction/exposure, an increase was observed in pro-inflammatory cytokines, such as IL-1, tumor necrosis factor-alpha (TNF-α), and IL-6, in systemic circulation and in brain tissue after periodontal infection [1,2,93]. Microglial activation was frequently reported; some studies suggest a shift toward a detrimental pro-inflammatory phenotype with chronic exposure. Long-term stimulation resulted in dysfunction of phagocytosis of amyloid-β, synaptic damage, and neuronal damage [28,94]. Activation of important inflammatory pathways, such as NF-κB, NLRP3 inflammasome, and STAT3 signaling, was common [19,93,100].

A significant amount of evidence showed that hallmark AD pathology may be promoted by periodontal infection in some animal models, consistently affecting amyloid-β and tau proteins. There was an augmentation of amyloid-β generation centrally and peripherally, and, according to some studies, macrophages and periodontal tissues contribute to the systemic amyloid load [23,92]. Also, tau hyperphosphorylation was reported in multiple studies, implicating signaling pathways including GSK3β signaling pathways, which are triggered by bacterial components like LPS and OMVs [8,19,21]. Other pathogens that seem to be involved are *Treponema denticola *[36,56,61,63], *Fusobacterium nucleatum *[54,101], *Prevotella nigrescens *[41], as well as *Porphyromonas gulae *[95]. These results indicate that periodontal disease has effects on major pathological characteristics of AD.

Neuroinflammation, amyloid deposition, and cognitive impairment associated with *P. gingivalis *were demonstrated to be induced independently by LPS and OMVs [7,8,19]. OMVs could readily penetrate biological barriers and transport pathogenic molecules to the brain, triggering immune responses and causing disease [19,27,92]. Breakage of the BBB was found to be a facilitating mechanism. Periodontal infection affected BBB permeability through augmented transcytosis and structural degeneration [9,99]. Oral bacteria’s extracellular vesicles were also discovered to penetrate the BBB and further impair its integrity, potentially creating a self-reinforcing cycle that may increase CNS exposure to inflammatory/toxic mediators [19,92].

New evidence brought to the fore the importance of the gut-brain axis in mediating oral dysbiosis effects [15,17,96]. Periodontitis was linked with changes in the composition of gut microbiota, in some animal studies, which may be influenced by antibiotic exposure, housing, and diet, which in turn results in systemic inflammation and deepens the AD pathology [15,17]. The transfer of oral microbiota was shown to aggravate cognitive impairment and amyloid deposition, suggesting that oral, gut, and brain systems are intricately related [15,96]. Metabolic comorbidities like obesity and diabetes were identified to enhance the impact of periodontal disease on AD progression [5,38]. Periodontitis was also demonstrated to impair the insulin signaling pathway and worsen insulin resistance, which leads to amplified amyloid and tau pathology [21,39]. These results imply a reciprocal connection where metabolic dysfunction and periodontal disease mutually enhance each other and result in a greater neurodegenerative risk [17,38].

A number of studies revealed that the production of amyloid-β might not be limited to the brain [23,24]. The response to bacterial infection by periodontal tissues and immune cells, especially macrophages, was found to cause amyloid-β [24,92]. This implies that peripheral sources can be a source of systemic amyloid, which can have an effect on brain pathology [23,99].

Experimental research examined different therapeutic options for infection-induced neurodegeneration [14,95]. Gingipain inhibitors demonstrated encouraging outcomes for bacterial load and neurodegenerative changes [6,14]. Anti-inflammatory agents, probiotics, bacteriocins (nisin), and immune modulation strategies were also used. The interventions showed a decrease in neuroinflammation, amyloid burden, and cognitive impairment in animal models [32,34,97].

This paper attempts to identify and summarize research that links periodontal disease with AD and summarizes findings suggesting consistent mechanistic patterns across several animal models [3,11,12]. The consistency across heterogeneous models suggests the hypothesis warrants further investigation [13,28,96]. *P. gingivalis *plays a prominent role in this issue; yet again, this does not happen coincidentally but consistently and through independent investigations [9,10,14]. This paper provides an interpretation of the discussed findings and an analysis of the mechanism implied by the existing evidence [4,96].

The most notable individual discovery among all the evidence collected in animal studies is the identification of* P. gingivalis* DNA and gingipains in postmortem AD brain tissue [14,92]. While confounding and reverse causality remain important considerations, reports of bacterial components in postmortem brain tissue add biological plausibility; however, causality remains unproven [4,96]. Neuroinflammation is no longer considered a mere by-product of amyloid deposition but rather a primary contributor to the pathogenesis of AD, in which peripheral systemic inflammation activates microglia to shift to a harmful pro-inflammatory phenotype [102]. With inhibition of the bacteria’s proteases showing positive effects on both neurological and biological parameters in animal models, one begins to suspect the presence of a mechanism that needs investigating [6,14]. Apparently, bacteria have a way of reaching the brain, where local damage caused by inflammation becomes difficult to resolve [9,10].

Here comes another notable factor: live bacteria are not necessary for the development of neurotoxicity [8,19]. LPS and OMVs secreted by *P. gingivalis*, under normal conditions, enter the brain and reproduce the hallmarks of AD alone: beta-amyloid production, hyperphosphorylation of tau protein, and measurable cognitive impairment [7,8,19]. Both LPS and OMVs activate the NLRP3 inflammasome and associated signaling pathways, leading to GSK3β-mediated hyperphosphorylation of tau protein and increased production of beta-amyloid [8,19,21].

A more general concern about these findings is their structural nature and requires discussion [9,99]. Indeed, the whole purpose of the BBB is to prevent what is happening inside of it. Under normal conditions, the barrier works well; nevertheless, the presence of* P. gingivalis* worsens things in several ways: first, by impairing transcytosis; second, by breaking down the structure of the barrier itself; and third, by making the barrier’s selective function progressively less effective [9,99]. Finally, vesicles from oral bacteria crossing the barrier not only fail to preserve it but actually help degrade it while passing [27,92]. A plausible self-reinforcing interaction was reported in some studies [9,96].

This is not a one-off event involving neuroinflammation but a condition [1,2,93]. Periodontal infection causes a prolonged increase in IL-1β, TNF-α, and IL-6, and the resultant microglial reaction is initially adequate [1,33]. Microglial activation may begin as an adaptive response, but chronic stimulation may impair clearance pathways, reducing the ability to clear amyloid-β, maintain synaptic function, and even start contributing to the very neuronal loss that they were destined to prevent [28,94]. *P. gingivalis *seems to have a specific effect on microglial phagocytic activity, which, if true, implies that the bacterium is not merely causing inflammation but actively impairing the major brain system responsible for handling its aftermath [18,94]. Oxidative stress compounds are further driven by NOX4, and at this point, there is no longer a distinction between inflammation and redox damage [30].

One thing that the AD field has been perhaps slow to consider is that amyloid might not necessarily be produced solely in the brain [23,24]. This is a question raised by the data reviewed here. Periodontitis increases amyloid-β in circulation [23,98]. Amyloid production in response to bacterial challenge is reinforced by macrophages and periodontal tissue [24,92]. The gums also have impaired processing of the amyloid precursor protein [37]. Taken individually, each of these observations is interesting. Combined, they pose a more clinically important prospect: that the oral cavity is an active player in systemic amyloid burden, not just a site of infection that coincidentally correlates with AD [4,96]. Should that be true in human populations, the consequences for modeling disease onset are substantial [12,13].

The gut-brain axis role in periodontal-Alzheimer interactions is still not completely understood [15,17,96]. A possible framework is the following: oral dysbiosis does not remain localized. It changes the composition of gut microbiota, and these changes have inflammatory effects, aggravating the pathology associated with AD [15,17]. This is no longer a bilateral affair between the mouth and the brain: there is a third component involved, and there is no clear understanding of how all three interact [17,96]. It becomes apparent that, in the case of gut homeostasis failure and increased permeability, the brain is also affected. The extent to which the neuroinflammatory load of AD can cause oral dysbiosis through this pathway is yet to be established, but it is a question worth considering [4,13].

Much more attention should be paid to metabolic comorbidities than is usually the case here [5,38,39]. Obesity and diabetes are not incidental traits of the patient population; they actively increase the neurodegenerative effects of periodontal infection [5,38]. Conversely, periodontitis interferes with insulin signaling and exacerbates insulin resistance, further aggravating amyloid and tau pathology [5,39]. A periodontal disease and type 2 diabetes patient is not navigating two separate conditions. They may interact synergistically, although the magnitude of that interaction in humans remains uncertain, with the possibility of contributing to neurological damage [5,38]. This has been slow to be reflected in clinical frameworks, and that is significant [38,39].

Loss of teeth is usually put in the perspective of a result of oral illness, rather than a precursor of a complication [44,46]. The framing is complicated by animal evidence. Tooth loss alone can induce hippocampal alterations, both structural and functional, indicating mechanical, somatosensory, and potentially neurotrophic mechanisms, which have attracted minimal research in the AD literature [42,43,46]. These findings are preclinical and should be interpreted cautiously [44,47]. But a question to be kept in mind is: does the oral-brain relation run even farther than the infection-based literature indicates [48,49]?

When the mechanisms given above are real, as the evidence suggests they are, then they are not merely intellectually interesting. They are actionable therapeutically [6,14]. The most apparent place to begin is with gingipain inhibition: find the virulence factor that continues to appear in the data and see what will change [14,95]. Preclinical results have been promising [14,95]. Other anti-inflammatory drugs like imipramine and microbiome-directed therapy with probiotics and bacteriocins like nisin have also been shown to have the ability to lower amyloid load, neuroinflammation, and cognitive impairment in experimental models [25,32,34]. Immune modulation through myeloid-derived suppressor cells may be the most provocative avenue of thought, since it shifts the focus to the host response per se, which may turn out to be where durable benefit is. Oral health interventions may represent a modifiable contributor worth studying in AD prevention/management, although human trials are necessary to support this theory [10,14].

Any of this would be a mistake, however, to treat as settled [11,13,14]. Nearly all of what is examined here is based on animal models, and that is a very real limitation [3,11,13]. Rodent models do not fully recapitulate the complexity and timeline of human AD. There is no standardization of the infection measures employed in studies [13,36]. Outcome measures vary [11,39]. It takes interpretive work to draw clean lines of inference across this literature that is ever credited to it. The truthful stance is that we have an argument of mechanically compelling persuasion constructed to a significant degree on preclinical underpinnings, and that does not equate to clinical evidence [3,12]. The prospective human data that now needs to be obtained includes longitudinal studies with careful periodontal characterization, validated cognitive endpoints, and long-term follow-up to capture a disease that is decades-long in onset. Much of that is yet to be done [13,50].

So what is our situation? The preclinical evidence suggests the association is unlikely to be purely incidental, but human causal inference remains to be established [14,20,96]. Bacterial invasion, neuroinflammation, barrier failure, vesicle toxicity, microbiome disruption, and metabolic amplification; these are not individual findings [5,9,17,19]. They create a consistent and structurally supportive image. The suggestion that oral health is an alterable risk factor of neurodegeneration is, at best, an understatement of what the data all indicate [5,12,14,20]. Human studies are still needed to close the evidentiary gap [13,50]. However, should they ratify what the preclinical literature already indicates is the case, the implications, in the way we prevent AD, in the way we treat it, and in the manner in which we structure the relationship between dentistry and neurology, are difficult to overestimate [13,14]. The brain and the mouth have been two realms treated as separate. This fact makes that divide seem increasingly arbitrary [14,96].

## Conclusions

Preclinical (animal) evidence supports plausible mechanistic links between periodontitis and AD. The translational gap requires human trials in order to be addressed. Microbes and virulence factors seem to be at the center of the activation and spread of neuroinflammatory and neurodegenerative pathways by periodontal pathogens. This relationship is mediated by multiple interconnected pathways, such as BBB malfunction, chronic neuroinflammation, amyloid and tau pathology, interactions between the microbiome, and metabolic dysfunction. Notably, these results indicate that periodontal disease may contribute to AD-like pathology in animal models and may represent a modifiable risk factor worth testing in human studies. Although the preclinical evidence is strong, translation to human disease remains a challenge. The use of animal models, diversity in experimental designs, and the absence of longitudinal human data limit the capacity to make concrete clinical inferences. However, the consistency of results across various studies demonstrates the potential significance of oral health in neurodegenerative disease. Future studies should be based on well-designed longitudinal studies and clinical trials encompassing standardized periodontal exposure definitions, APoE/vascular/metabolic confounders, validated cognitive outcomes, and adequate follow-up.
